# Supplementary material for: Beneficial Shifts in Gut Microbiota by Lacticaseibacillus rhamnosus R0011 and Lactobacillus helveticus R0052 in Alcoholic Hepatitis
Source: Microorganisms. 2022 Jul 21;10(7):1474. doi: 10.3390/microorganisms10071474 (PMC9319967; doi:10.3390/microorganisms10071474)
Supplement: Supplementary file 1 [file microorganisms-10-01474-s001.zip › microorganisms-1814878-supplementary.pdf]

microorganisms

Supplementary materials

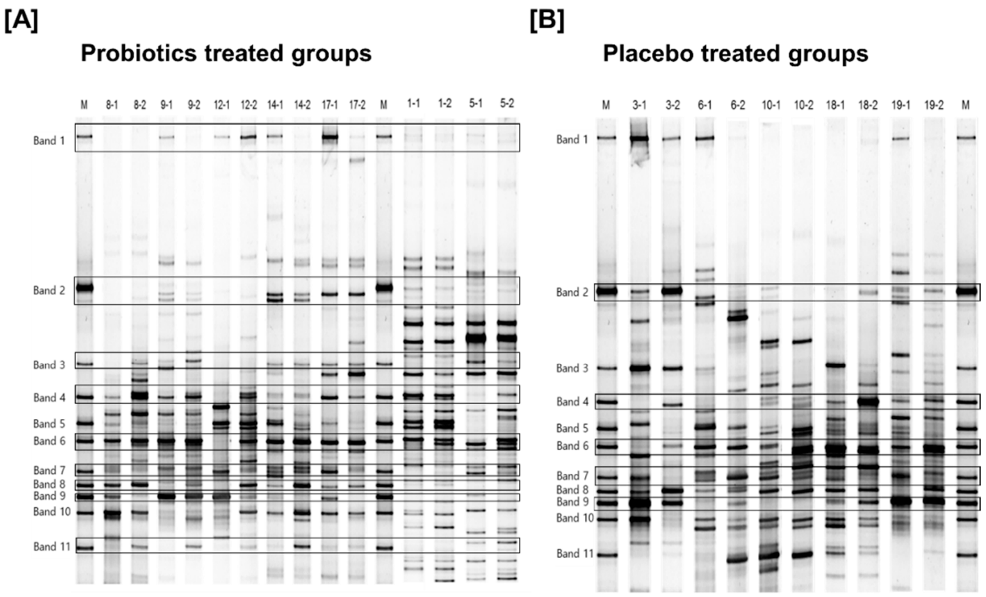

[C]

List of bands identified in probiotic and placebo group

Band	Closest relatives	Similarity
1	*Bacteroides fragilis*	100%
2	*Bacteroides* sp.	100%
3	*Parabacteroides distasonis*	100%
4	*Eubacterium eligens*	100%
5	*Fusobacterium mortiferum*	100%
6	*Faecalibacterium prausnitzii*	100%
7	*Clostridiales* sp.	100%
8	*Veillonella dispar*	100%
9	*Escherichia coli*	100%
10	*Veillonella parvula*	100%
11	*Enterobacter* sp.	100%

- Sample information (16 patients_38 samples)

Figure S1. Denaturing gradient gel electrophoresis (DGGE) analysis of fecal samples of patients before and after treatment [A] Probiotic treated groups (n=14). [B] Placebo treated groups (n=10). [C] List of bands identified in probiotic and placebo group.

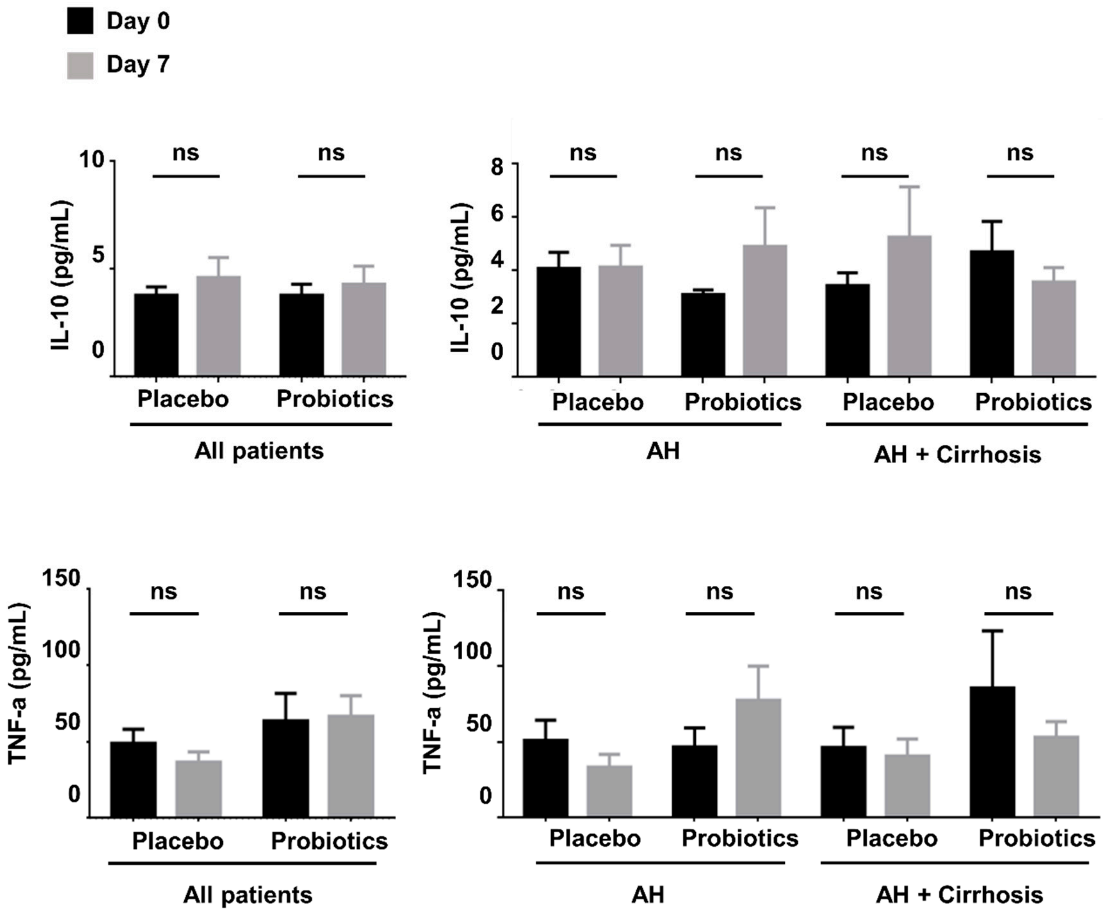

Figure S2. Serum measurement of cytokines level in patients before and after treatment. [A] Interleukin-10 level. [B] Tumor necrosis factor-a level.

[A]

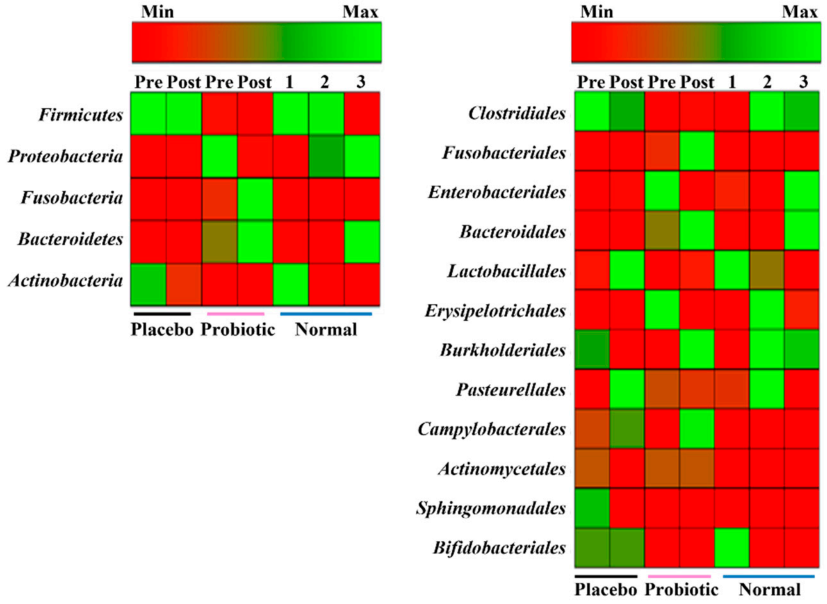

Figure S3. Microbial change after treatment. [A] Heatmaps depicting changes in phylum and class level after placebo and probiotic treatment. [B] Changes in relative abundance of species in probiotic and placebo groups after 7 days treatment.
